# Redundancy and Cooperativity in the Mechanics of Compositely Crosslinked Filamentous Networks

**DOI:** 10.1371/journal.pone.0035939

**Published:** 2012-05-09

**Authors:** Moumita Das, D. A. Quint, J. M. Schwarz

**Affiliations:** 1 Department of Physics and Astronomy, Vrije Universiteit, Amsterdam, The Netherlands; 2 Physics Department, Syracuse University, Syracuse, New York, United States of America; Dalhousie University, Canada

## Abstract

The cytoskeleton of living cells contains many types of crosslinkers. Some crosslinkers allow energy-free rotations between filaments and others do not. The mechanical interplay between these different crosslinkers is an open issue in cytoskeletal mechanics. Therefore, we develop a theoretical framework based on rigidity percolation to study a generic filamentous system containing both stretching and bond-bending forces to address this issue. The framework involves both analytical calculations via effective medium theory and numerical simulations on a percolating triangular lattice with very good agreement between both. We find that the introduction of angle-constraining crosslinkers to a semiflexible filamentous network with freely rotating crosslinks can cooperatively lower the onset of rigidity to the connectivity percolation threshold—a result argued for years but never before obtained via effective medium theory. This allows the system to ultimately attain rigidity at the lowest concentration of material possible. We further demonstrate that introducing angle-constraining crosslinks results in mechanical behaviour similar to just freely rotating crosslinked semflexible filaments, indicating redundancy and universality. Our results also impact upon collagen and fibrin networks in biological and bio-engineered tissues.

## Introduction

The mechanical response of most cells arises from the mechanics of its cytoskeleton, a polymeric scaffold that spans the interior of these cells, and its interaction with the extra-cellular environment. The cytoskeleton is made up of complex assemblies of protein filaments crosslinked and bundled together by a variety of accessory proteins. For example, there are approximately 23 distinct classes of accessory proteins such as fascin, a-actinin, and filamin A [1] that crosslink filamentous-actin (F-actin), a major component of the cytoskeleton responsible for the mechanical integrity and motility of cells. Given the multitude of crosslinkers, several natural questions arise: Are the different types of crosslinkers redundant, or do they each serve specific functions? Do they act independently or do they cooperate to allow the cell to optimize its mechanical response? What are the consequences of their mechanics for the mechanical integrity and response of the cell?

To begin to answer these questions, a mutation study of *dictyostelium discoideum* cells lacking a particular actin crosslinker can still grow, locomote, and develop, though with some defects, thereby suggesting at least partial redundancy in the crosslinker’s mechanical function [2]. On the other hand, two types of crosslinkers working cooperatively has been demonstrated in stress fibers crosslinked with the actin binding proteins (ABP) 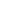-actinin and fascin, where stress fibers containing both 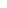-actinin and fascin were more mechanically stable than stress fibers containing only 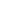 -actinin or fascin [3]. It could also be the case that different crosslinkers work independently of one another such that the dominant crosslinker dictates the mechanical response of the network [4]. Given these various possibilities, how the cell uses different crosslinking proteins to optimize for certain mechanical characteristics is an important open issue in cytoskeletal mechanics.

Here, we theoretically address the interplay between crosslinkers by studying a model network of semiflexible actin filaments crosslinked with two types of flexible crosslinkers. We first study the mechanical properties of the model network with one type of crosslinker and then introduce the second type and look for mechanical similarities and differences with the original network. As for the two types of crosslinkers, we consider crosslinkers that allow the crossing filaments to rotate energy-free (freely-rotating crosslinks) and crosslinkers where there exists a finite energy cost to rotating two crossing filaments with respect to each other (angle-constraining crosslinks). While the work presented here is a parameter study, one of the parameters being the energy cost for rotating two crosslinked filaments with respect to each other, it is useful to consider possible candidate crosslinkers for the sake of concreteness. The ABP 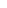-actinin is a candidate for the freely-rotating crosslinker. Recent optical trapping studies demonstrate that two filaments bound by 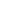-actinin can rotate easily [5]. As for an example of the latter, an angle-constraining crosslinker, we propose filamin A (FLNa) as a possible candidate. While indeed both alpha-actinin and FLNa are flexible crosslinkers [6]–[8], a recent model for FLNa binding in the network regime consists of FLNa binding two actin filaments at a reasonably regular angle of ninety degrees [9], [10]. This model suggests that FLNa crosslinking can be modeled as an angular spring, with a small but finite stiffness, connecting the two actin filaments. It turns out that the results presented here will support this recent model. We also note that here we do not take into account the hinging of each molecular arm of FLNa binding to each actin filament [11], nor its unfolding occuring at large mechanical stresses [9]–[12], since we seek to understand fully the mechanics in the network regime with small applied strains first.

The introduction of angle-constraining crosslinkers also opens the door to mechanical modelling of Arp2/3 as a crosslinker in the actin cortex. To date, Arp2/3′s role as an F-actin nucleator has been emphasized in lamellipodia formation [13], [14]. However, its role in constraining the angle between the mother and daughter filaments to roughly seventy degrees [15] is presumably also important for lamellipodia mechanics. It would be interesting to explore Arp2/3′s mechanical role in lamellipodia formation, which may be just as important as its nucleation role. In addition, accounting for the mechanics of angle-constraining crosslinkers is necessary for realistic modeling of collagen and fibrin networks. These networks often show a branched architecture with fairly regular angles, i.e. filaments reaching across three legs in 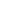 shaped junctions [16], [17]. Both collagen and fibrin networks may ultimately play an important role as biopolymeric scaffolds in tissue engineering [18], [19].

In studying the mechanical properties of compositely crosslinked filamentous networks, we focus on the onset of mechanical rigidity as the filament concentration is increased above some critical threshold. This onset is otherwise known as rigidity percolation [20]–[26]. Above this critical threshold, both experiments and theoretical studies of F-actin networks have observed distinct mechanical regimes. For dense, stiff networks the mechanical response is uniform, or affine, and the strain energy is stored predominantly in filament stretching modes. While for sparse, floppy networks one finds a non-affine response dominated by filament bending where the observed mechanical response of the network is inhomogeneous and highly sensitive to the lengthscale being probed [27]–[33]. Recent theoretical studies have reported that there also exists a *bend-stretch* coupled regime for intermediate crosslinking concentrations and filament stiffnesses [34], [35]. We investigate these different mechanical regimes in our compositely crosslinked networks. While considerable progress has been made in understanding the mechanics of single component and composite cytoskeletal networks crosslinked by one type of crosslinker [27]–[33], [36]–[38], compositely crosslinked networks are only beginning to be explored experimentally [4], [39] and there exists little theoretical understanding of their synergistic mechanical response.

The most remarkable findings of our work are the following. We demonstrate both cooperative and redundant mechanics in compositely crosslinked filament networks that allow the system to be simultaneously adaptable and robust. To this end we use analytical and computational methods to study a network of filaments, with a broad length distribution, arranged on a two dimensional lattice. In particular, we show that freely-rotating and angle-constraining crosslinkers, even when the cost of constraining angles is very small, can cooperate to enable the network to tune its mechanical rigidity at a given filament concentration and given total crosslinker concentration. We also show that the mechanics of stiff filament networks, whether present with one type of crosslinkers or multiple types, has some universal features in the form of distinct mechanical regimes that are governed by the stretching or bending elasticity of the filaments or a combination of the two, suggesting a built-in redundancy. Finally, we demonstrate that the threshold for rigidity in compositely crosslinked networks can essentially be as low as the filament concentration required to form a geometrically percolating structure, a result conjectured over two decades ago but never before proved in an effective medium theory.

To compare our results with the mechanics of networks of organic polymers such as polyacrylamide, we also investigate the interplay of two types of crosslinkers for networks made of flexible filaments. While most biological filaments including F-actin, collagen and fibrin are semiflexible, i.e. their elasticity is intermediate between that of rigid rods and flexible rubber-like polymers, many organic polymers, including polyacrylamide, are flexible and can be modeled as entropic springs. Typically the rheology of single flexible polymers is very different from that of semiflexible filaments, with only the latter showing strain stiffening at small strains [40]. We demonstrate that flexible filament networks with angle-constraining crosslinks, at small enough distances between crosslinks, can mimic linear elastic behavior very similar to semiflexible filament networks with freely-rotating crosslinks.

## Methods

**Figure 1:**
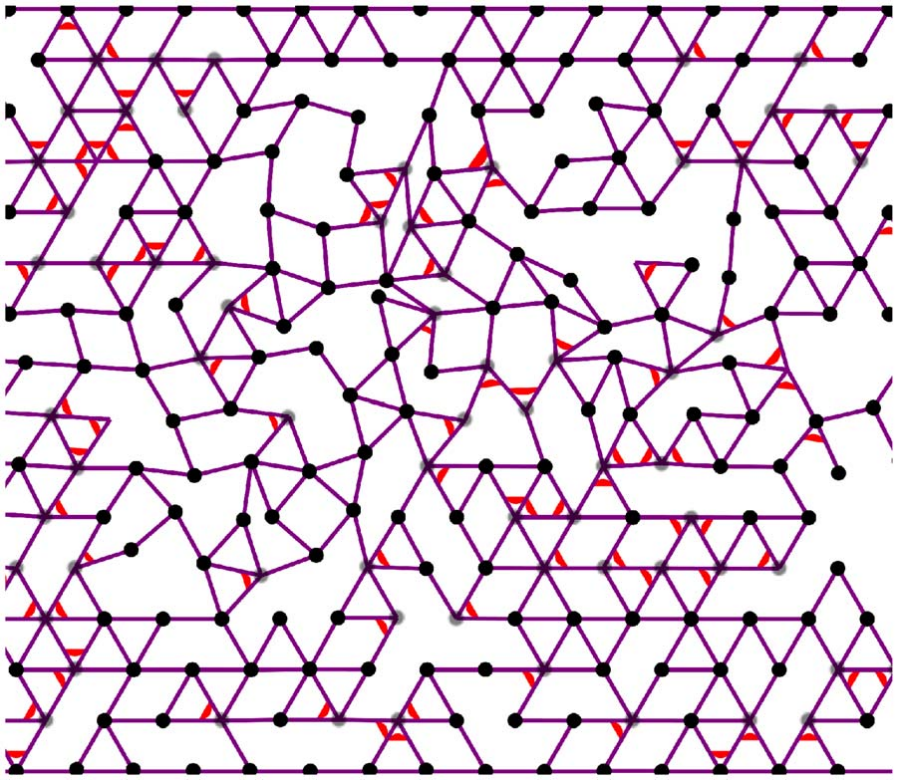
Deformed configuration a compositely crosslinked semiflexible filament network with 2.7 percent strain. The bond occupation probability is 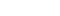 and angle-constraining crosslinker occupation probability is 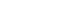 The purple lines denote semiflexible filaments, the red arcs denote angle-constraining crosslinks, the black circles represent nodes where all crossing filaments are free to rotate around that node, while the grey circles denote nodes where some of the crossing filaments are free to rotate around that node. The absence of a black or grey circle denotes a node where no free rotations are possible. The filament bending stiffness relative to stretching stiffness 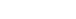 and the stiffness of angular crosslinks relative to stretching stiffness 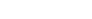

To create a disordered network of crosslinked filaments, we arrange infinitely long filaments in the plane of a two-dimensional triangular lattice. The filaments are given an extensional spring constant 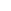 and a filament bending modulus 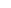 We then introduce finite filament length 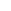 into the system by cutting bonds with probability 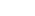 where 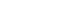 with no spatial correlations between these cutting points. The cutting generates a disordered network with a broad distribution of filament lengths. When two filaments intersect, there exists a freely-rotating crosslink preventing the two filaments from sliding with respect to one another. Next, we introduce angular springs with strength 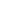 between filaments crossing at 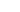 angles with a probability 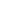 where 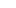 denotes non-collinear. These angular springs model the second type of crosslinker. When 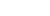 all 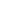 crossings between filaments are occupied by an angle-constraining crosslinker such that 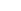 is a measure of the concentration of the second type of crosslinker. See Fig. 1 for a schematic.

We study the mechanical response of this disordered network under an externally applied strain in the linear response regime. For simplicity we set the rest length of the bonds to unity. Let 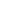 be the unit vector along bonds and 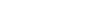 the strain on the bond 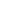 For small deformation 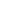 the deformation energy is
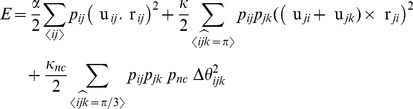
(1)where 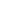 is the probability that a bond is occupied, 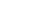 represents sum over all bonds and 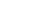 represents sum over pairs of bonds sharing a node. The first term in the deformation energy corresponds to the cost of extension or compression of the bonds, the second term to the penalty for the bending of filament segments made of pairs of adjacent collinear bonds, and the last term to the energy cost of change in the angles between crossing filaments that meet at 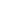 angle. Furthermore, for small deformations 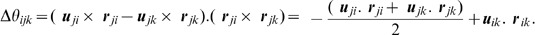 It is straightforward to see that the angular spring 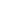 between 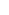 and 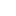 will contribute to an effective spring in parallel with 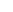, giving rise to an enhanced effective spring constant 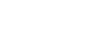 Note that in the limit 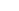 becomes infinite, the crosslinker is completely rigid.

### Effective Medium Theory

We study the effective medium mechanical response for such disordered networks following the mean field theory developed in [23], [24] for central force networks and [33] for filament bending networks. The aim of the theory is to construct an effective medium, or ordered network, that has the same mechanical response to a given deformation field as the depleted network under consideration. The effective elastic constants are determined by requiring that strain fluctuations produced in the original, ordered network by randomly cutting filaments and removing angular springs vanish when averaged over the entire network.

To perform the disorder averaging, since the stretching of filaments is defined in terms of spring elasticity of single bonds 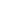 the disorder in filament stretching is given by 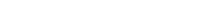 Filament bending, however, is defined on pairs of adjacent collinear bonds with the normalized probability distribution 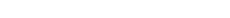 Similarly, for the angular springs, which is also defined on pairs of bonds, the normalized probability distribution is given by 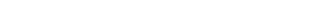 Using this disorder averaging, we derive the effective medium elastic constants as functions of 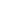 and 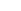 as shown in the Supporting Information File S1. The effective medium filament stretching modulus 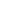 is obtained by solving
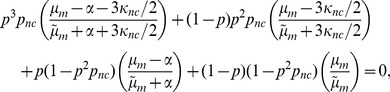
(2)where 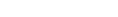 The effective medium elastic moduli for filament bending 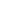 and stiffness of the angular springs 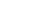 are given by



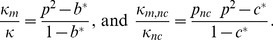
(3)The constants 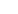 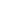 and 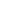 are dimensionless variables that describe the network contribution to 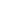 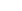 and 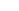 respectively. They can be expressed in terms of the components of the dynamical matrix for the ordered network, as described in the Supporting Information File S1, as 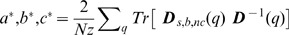 where the subscripts 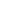 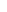 and 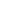 stand for filament stretching, filament bending and bending of angles between filaments crossing at 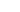 angles. The sum is over the first Brillouin zone and 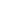 is the number of nearest neighbors for each crosslink or node. The dynamical matrix is determined by the force constants between a set of reference nodes (here crosslinks) in the ordered network (lattice)and all their neighbors, and contains all the information that determines the displacements in the ordered network. Also, by definition, 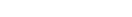 where 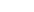 is the dimensionality of the system. Note that at the rigidity percolation threshold 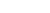 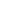 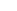 and 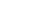 vanish, giving 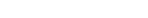 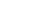 and 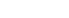


### Numerical Simulations

Simulations were carried out on a triangular lattice with half periodic boundary conditions along the shear direction for the energetic terms whose small deformation limit is given in Eq. (1). Networks were constructed by adding bonds between lattice sites with probability 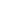 Next, a shear deformation was applied to the two fixed boundaries of magnitude 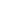 The lattice was then relaxed by minimizing its energy using the conjugate gradient method [41] allowing the deformation to propagate into the bulk of the lattice. Once the minimized energetic state was found within the tolerance specified, in this case the square root of the machine precision 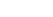 the shear modulus was then measured using the relation, 
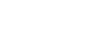
 using small strains 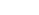 with 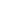 denoting the system length and 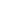 denoting the area of the unit cell for a triangular lattice which is equal to 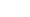 in our units. System sizes 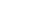 (shown unless otherwise specified) and smaller were studied. Sample averaging was performed such that the curves are sufficiently smooth. For example, for 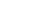 an average over 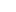 runs was performed.

## Results

### Mechanical Integrity as Measured by the Shear Modulus

We first investigate how the mechanics of the network depends on the average length of the filaments and the concentration of crosslinkers. The average filament length increases with the probability 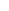 that a bond is occupied as 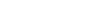 while the concentration of the crosslinkers that tend to constrain the angle between crossing filaments is simply 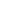 To determine the filament lengths and crosslinker concentrations at which the network attains mechanical integrity or a finite shear modulus and how the network rigidity changes thereafter, we focus on the shear modulus 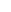 as a function of 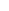 and 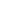


**Figure 2:**
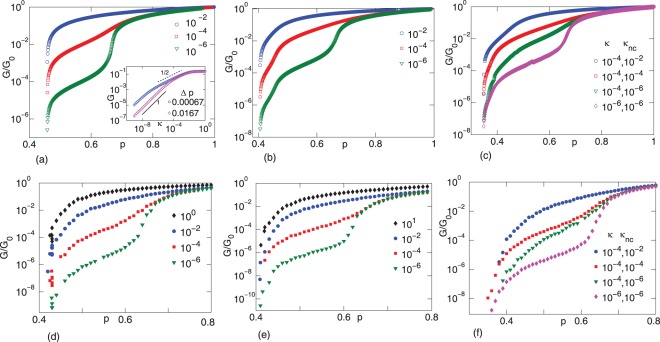
The shear modulus 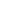 normalized by its value for the corresponding undiluted network 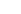 as a function of occupation probability 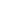 Semiflexible networks with freely-rotating crosslinks are depicted in (a) and (d). Flexible networks with freely-rotating and angle-constraining crosslinks are shown in (b) and (e), and semiflexible networks with both crosslinkers are depicted in (c) and (f). In the latter two cases, all freely-rotating crosslinks with filaments crossing at 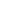 have been replaced with angle-constraining crosslinks 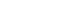 The legends in (a),(b),(d) and (e) represent different values of the bending stiffness of filaments 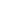 while the legends in (c) and (f) represent different 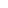 and stiffness of angle-constraining crosslinkers 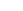 The top panels show results from the effective medium theory and bottom panels show results from the simulations. Inset in (a) shows the three mechanical regimes for the freely-rotating crosslinked semiflexible network (the legend shows the separation 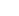 from the isostatic point), where the shear modulus 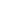 is independent of 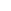 scales as 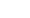 and as 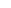

On a triangular lattice, networks made solely of Hookean springs lose rigidity at a bond occupation probability around 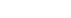 [22]–[24], [42]. This result corresponds to the central force isostatic point at which the number of constraints is equal to the number of degrees of freedom on average. In contrast, networks made of semiflexible filaments become rigid at a smaller 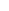 due to extra constraints placed on the system via filament bending. For semiflexible networks with freely-rotating crosslinks, our effective medium theory shows that the shear modulus, 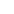 approaches zero at 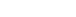 as shown in Fig. 2 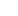. This result is in good agreement with our simulation results yielding 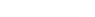 and previous numerical results [35]. See Fig. 2 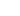. A different formulation of mean field theory yields 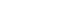 [35]. By replacing freely-rotating crosslinks between filaments crossing at 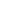 with angle-constraining crosslinkers (with a resting angle of 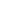), the rigidity percolation threshold is lowered. Our EMT yields 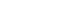 and our simulations yield 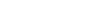 for 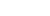 when all freely-rotating crosslinks between filaments crossing at 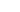 have been replaced with angle-constraining ones (Fig. 2 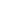 and 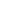). The cooperative mechanical interplay between these crosslinks and their interaction with filaments allows the network to form a rigid stress-bearing structure at remarkably low crosslinking concentrations, almost immediately after it attains geometric percolation, 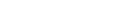 which agrees with an argument by Kantor and Webman [43]. For flexible filament networks, introducing angle-constraining crosslinkers also lowers the rigidity percolation threshold as compared to the isostatic point with the network attaining rigidity at 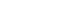 for our EMT and 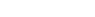 in the simulations, again, for 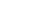 ((Fig. 2 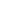 and 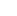). Incidentally, our result agrees very well with a previous simulation [44].

**Figure 3:**
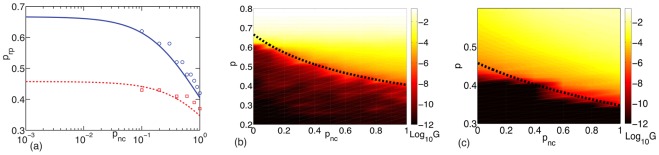
The presence of angular constraints allows compositely crosslinked networks to have a finite rigidity even for small concentration of filaments. Figure (a) shows how the rigidity percolation threshold can be continuously lowered by increasing the probability (concentration) of angular springs for flexible (solid, blue) and stiff (dashed, red) networks. The lines correspond to the effective medium theory and the symbols to the numerical simulation. Figures (b) and (c) show the shear modulus (in logarithmic scale described by the colorbar) as a function of 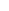 and 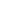 for flexible networks (b) and semiflexible networks (c). The parameter values studied are (b) 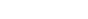 and (c) 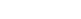 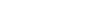 The black dashed lines in (b) and (c) correspond to the effective medium theory prediction of the rigidity percolation threshold. For the flexible networks, 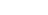 while for the semiflexible networks, 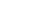

In addition to examining the rigidity percolation threshold for 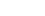 for both compositely crosslinked semiflexible and flexible networks, we also compute analytically and numerically how 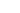 changes with 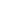 for both types of networks. See Fig. 3 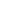. As 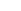 is increased from zero to unity such that an increasing number of angle-constraining crosslinkers are introduced into the system while keeping the total crosslinker concentration fixed, the rigidity percolation threshold, 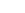 is lowered continuously with good agreement between our analytical and numerical calculations. Concomitantly, there is a substantial increase in the shear modulus with increasing 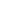 particularly for filament concentrations near the rigidity percolation threshold. See Figs. 3 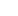 and 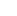. For example, according to Fig. 3 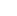, for 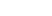 the shear modulus increases by approximately two orders of magnitude as 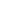 is increased from zero to unity. The introduction of the second type of crosslinker allows for a more mechanically versatile system.

Now let us review the behavior of the shear modulus as a function of 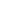 for a semiflexible network with just freely-rotating crosslinks 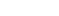 See Figs. 2 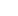 and 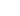. Just above the rigidity percolation threshold, we find a bending-dominated regime for sparse networks with the shear modulus eventually crossing over to a stretch dominated affine regime at higher filament concentrations. The purely stretch dominated regime is represented by the macroscopic shear modulus 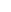 scaling linearly with 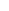 with a very small slope, and here 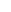 is governed solely by the filament stretching elasticity 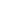 In the purely bend dominated regime, on the other hand, the network is highly floppy and 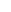 decreases rapidly as 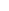 is lowered, and is controlled by the bending stiffness 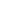 of the filaments, as observed previously in [27]–[29], [33], [35]. The bend-stretch coupled regime, on the other hand, is characterized by a shear modulus that is a generalized average of the stretch and bend elasticity of the filaments, i.e. 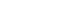 (where 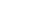), as discovered for filamentous networks based on the Mikado model in Ref. [34], and more recently found on a diluted triangular lattice in Ref. [35]. As seen in Ref. [35], for 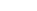 both the effective medium theory and the simulations yield such a bend-stretch coupled regime, which is indicated by an inflection in 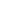 as a function of 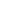 observed most clearly for 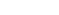 (with 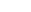) and a value of 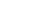 just below the isostatic point (inset in Fig. 2 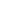).

The three mechanical regimes discussed above are robust and observed for both compositely crosslinked flexible and semiflexible filament networks. See Figs. 2 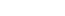, and 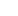. Regarding the bend-stretch coupled regime, for the flexible filament networks, this regime occurs for 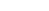 i.e. 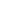 replaces 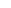 For semiflexible filament networks, on the other hand, as long as 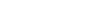 the bend-stretch coupled regime is robust (for fixed 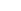). In contrast, for 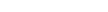 the angle-constraining crosslinker suppresses the bend-stretch coupled regime and enhances the shear modulus to that of an affinely deforming network (for fixed 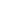). The mechanics of the network has been altered with the introduction of the second type of crosslinker in this range of the parameter space.

### Non-affinity Parameter

In dense or stiff networks that deform uniformly or affinely, one can use an affine formulation of continuum elasticity to describe and understand the mechanical response of the network anywhere and at any lengthscale in the system which is sufficiently larger than the crosslinking distance. However, for very sparse networks deep in the non-affinely deforming regime, the mechanical response in the network is no longer uniform or affine due to large local, quenched spatial variations in the elastic response of the network and is very sensitive to the lengthscale being probed. Thus, an important parameter used to determine the lengthscale above which an affine description can ultimately be formulated is the degree to which the network deforms non-affinely. Experimental and computational measures of this parameter require studying the network strain field, and quantitatively analyzing the degree of non-affinity.

To investigate how the interaction of the two types of flexible crosslinkers affects the affine and non-affine mechanical regimes in compositely crosslinked networks, we numerically study a quantitative measure for the degree of non-affinity 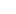 defined in Ref. [35] as:
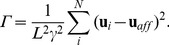
(4)


**Figure 4:**
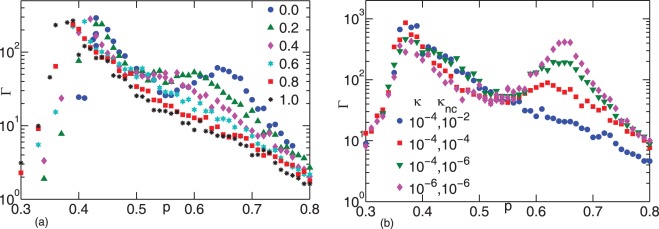
The non-affinity parameter 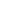 as a function of occupatio probability 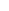 for semiflexible networks with both types of crosslinkers. In (a) we show the effect of replacing freely-rotating crosslinkers between filaments crossing at 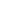 with angle-constraining ones, as denoted by 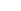 (whose values are shown in the legend), for 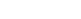 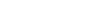 while in (b) we show the effect of changing their stiffness 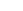

The above non-affinity parameter can also be interpreted as a measure of the proximity to criticality, diverging at a critical point as one approaches infinite system size. Two peaks in 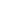 have recently been numerically observed in freely-rotating crosslinked semiflexible networks [35]. The first peak occurs at the rigidity percolation threshold, while the second peak occurs near the central force isostatic point. For our compositely crosslinked semiflexible newtorks, we find that 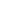 develops a peak at the rigidity percolation threshold, which progressively moves to smaller values of 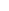 as the concentration of angular crosslinkers 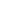 is increased (Fig. 4 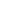). A second peak develops near the isostatic point for 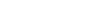 as seen in Fig. 4 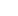. As both the collinear and non-collinear bending stiffnesses tend to zero, the network mechanics approaches that of a central force network, and the second peak in 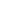 at the isostatic point becomes increasingly more pronounced.

On the other hand, this second peak can be suppressed by increasing 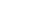 (Fig. 4 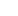), or by increasing 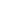 (Fig. 4 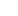) even for very small values of 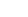 This further corroborates that adding angle-constraining crosslinkers to non-affine networks can suppress non-affine fluctuations, provided they energetically dominate over filament bending. The reason for this suppression can be understood by considering the effect of adding a constraint which prohibits the free rotation of crossing filaments. As the concentration of these non-collinear crosslinks 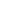 is increased (at fixed avg. filament length) microscopic deformations will become correlated. The lengthscale associated with this correlation will increase on increasing either 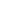 or 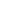 and will eventually reach a lengthscale comparable to the system size even at 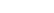 at large enough concentration and/or stiffness of the angular springs. As a result, the mechanical response of the network will approach that of an affinely deforming network. Upon decreasing the value of 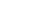 relative to 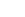 we again recover the second peak because energetically the system can afford to bend collectively near the isostatic point.

Note that the disorder used in this model leads to a broad distribution of filament lengths, which could imply the presence of a significant number of filaments spanning lengthscales comparable to the system size, when 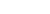 This can cause a suppression of the bending dominated and bend-stretch coupled mechanical regimes for sufficiently stiff filaments, as previously observed for freely-rotating crosslinked semiflexible networks [45]. Indeed, in our compositely crosslinked semiflexible networks, both regimes are distinctly observed only for networks made of filaments with very small bending rigidities. Instead, if the distribution of filament lengths is narrower, one should observe the bend dominated and bend-stretch coupled response at the same average filament length, for comparatively stiffer filaments.

### Scaling Near the Isostatic Point

Finally, we quantify the similarity in mechanics between freely-rotating crosslinked semiflexible networks and compositely crosslinked flexible networks with a scaling analysis. Scaling analysis helps identify the parameters in a system that control its behavior, particularly near a phase transition, and can identify universal features of phase transitions that appear in very different types of systems. Such analysis has been previously successfully used to infer the dominant material parameters that govern the mechanical response of filamentous networks based on the Mikado model [34] near the rigidity percolation transition and lattice-based disordered filamentous networks [35], [46] near the isotatic point.

**Figure 5:**
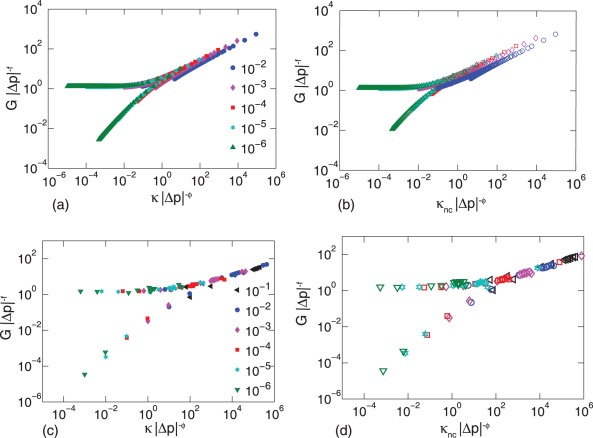
Scaling analysis of network mechanics near the isostaticity transition. The shear modulus 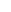 scales with 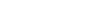 and 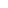 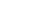 as 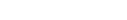 close to isostaticity. The effective medium theory predicts mean field exponents 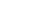 and 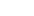 for both semiflexible networks with freely-rotating crosslinkers (a) and compositely crosslinked flexible networks (b), while simulations predict 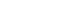 and 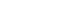 for semiflexible networks with freely-rotating crosslinkers (c) and 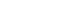 and 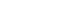 for compositely crosslinked flexible networks (d). In (a) and (c) we vary the stiffness of the filaments 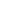 (represented by the legends) and in (b) and (d) we vary the stiffness of the angular constraints 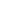 (symbols represent the same values as shown by the legends in (a) and (c) respectively).

To examine the robustness of the networks under consideration, we examine the scaling of the shear modulus 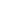 near the central force isostatic point with 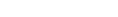 following the approach used in Ref. [35] for freely-rotating crosslinked semiflexible networks. For 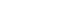 (or 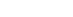), the shear modulus scales as 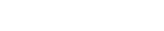 (or 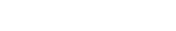) [35]. For both 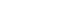 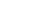 and 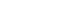 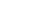 the EMT predicts 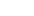 and 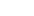 as shown in Figs. 5 (a) and (b), indicating that both types of networks demonstrate redundant, or generic, mechanics. To compare the EMT results with the simulations, we use the position in the second peak in 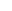 to determine the central force percolation threshold, 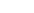 and then vary 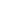 and 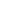 to obtain the best scaling collapse. For case (a), 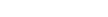 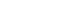 and 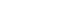 For case (b), 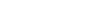 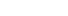 and 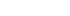 Both sets of exponents are reasonably consistent with those found in Ref. [35] for a semiflexible network with freely-rotating crosslinks only. Preliminary simulations for compositely crosslinked semiflexible networks indicate that the shear modulus scales as 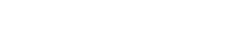 also with a similar 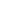 and a similar 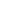 with 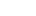 It appears that networks with both stretching and bending interactions (collinear and/or noncollinear) exhibit the same scaling, which provides for important evidence for a possible non-affine continuum description.

## Discussion

Since crosslinking of the actin cytoskeleton is done by a number of different crosslinkers, a natural question to ask is how does the possible interplay between the different types of crosslinkers affect the mechanics of the actin cytoskeleton? Do the two types of crosslinkers interact cooperatively in the sense that the introduction of a second type of crosslinker to a network crosslinked with one type allows the network to tune its mechanical response and enhance its ability to transmit forces? Or, does the compositely crosslinked network exhibit similar mechanical properties to networks crosslinked by a single type in which case the second (or even third) type of crosslinker is redundant?

To begin to address the interplay between different types of crosslinkers in the actin cytoskeleton, we introduce a model filamentous network composed of filaments randomly occupying an underlying triangular lattice. These filaments have both a bending rigidity 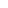 and a stretching stiffness 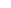 Wherever two filaments cross, there exists a freely-rotating crosslinker between them such that the two filaments cannot slide with respect to each other, only rotate with zero energy cost. We introduce a second type of flexible crosslinker in which there does exist an energy cost for rotating two filaments with respect to each other with rotational stiffness 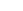 otherwise known as angle-constraining crosslinkers.

To summarize our results, we find:

1. If we begin with a purely freely-rotating crosslinked network and replace freely-rotating crosslinks between filaments crossing at 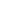 with angle-constraining crosslinkers, the minimum average filament length necessary to attain mechanical rigidity is lowered for both semiflexible and flexible networks. In other words, the composite system can more readily transmit forces at a given average filament length and given total crosslinker concentration than the purely freely-rotating one, hence the two types of crosslinkers work cooperatively. In both semiflexible and flexible networks, this decrease is independent of the energy scale of the crosslinker, however, for semiflexible networks, the rigidity threshold can be pushed down to the geometric percolation threshold–the lowest possible average filament length required to transmit forces–a finding which has important mathematical and biology implications. Moreover, depending on the parameters, the shear modulus can increase by several orders of magnitude with the addition of the second crosslinker all while keeping the filament concentration and total crosslinker concentration fixed.
2. The second interplay between the two crosslinkers depends on the ratio of the energy scale of the angle-constraining crosslinker to the filament bending energy. For very soft filaments 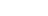 the freely-rotating semiflexible filament system exhibits large non-affine fluctuations near a particular average filament length known as the isostatic point. Upon addition of the angle-constraining crosslinkers that enforce these constrains tightly 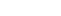 the non-affine fluctuations near this point become suppressed and the mechanics of the angle-constraining crosslinker dominates the system. Once again, with a small change in concentration of the second crosslinker while keeping the total crosslinker concentration fixed, the mechanical response of the network is changed dramatically resulting in a cooperative behaviour.
3. We demonstrate that singularly crosslinked and compositely crosslinked filamentous networks share some important, generic properties. In particular, all three networks studied here (freely-rotating crosslinked semiflexible networks and compositely crosslinked semiflexible and flexible networks) have three distinct mechanical regimes as a function of the average filament length: a regime dominated by the stretching elasticity of filaments, a regime dominated by the bending elasticity of filaments and/or stiffness of angle-constraining crosslinkers, and an intermediate regime which depends on the interplay between stretching and bending. We further show that for networks of soft filaments crosslinked with flexible angle-constraining crosslinkers (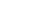 and 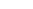) that the non-affine fluctuations near the isostatic point (the onset of rigidity in freely-rotating crosslinked flexible networks) remain large. The same is the case for semiflexible networks with freely-rotating crosslinks even with the addition of the angle-constraining crosslinkers. In addition, the scaling exponents near this regime also appear to be independent of the type of network, again, encouraging a universal theoretical framework for filamentous network mechanics with stretching and bending interactions and suggesting a built-in redundancy.

In conclusion, we have discovered both cooperative and redundant mechanical effects in compositely crosslinked filament networks such that this generic system is simultaneously adaptable and robust. We now discuss the implications of our results for various filamentous networks.

### Rigidity Percolation

In the case of the compositely crosslinked semiflexible filament networks, we demonstrate using effective medium theory and numerical simulations that the threshold for rigidity can be essentially as low as the filament concentration required to form a geometrically percolating structure. Certainly, the existence of a spanning cluster is the lower bound for the transmission of forces. Several decades ago it was argued that networks with angular interactions should exhibit a rigidity percolation threshold essentially equal to that of the geometric percolation threshold. While an earlier effective medium theory for a related model with stretching and bond-bending interactions obtained a rigidity threshold lower than the geometric percolation threshold as reported in Ref. [23] (an impossibility as acknowledged by the authors), our analytical calculation is the first effective medium theory result supporting the argument made some twenty years earlier, thereby accurately extending the reach of effective medium theory to angular (three-body) interactions. It would be interesting to try to extend our effective medium theory to include four-body interactions, which may become relevant for the nonlinear strain regime where longitudinal (stretching) and transverse (bending) displacements become coupled.

A new critical regime has recently been found in freely-rotating crosslinked semiflexible networks near the isostatic point [35]. This new regime is driven by an increase in nonaffine fluctuations. We find that this new regime extends to compositely crosslinked flexible networks. Preliminary data suggests the same scaling extends to compositely crosslinked semiflexible networks as well. Therefore, this new scaling regime more broadly applies than initially anticipated. It may be that as long as the system contains both two-body (stretching) and three-body (bending) interactions such a regime should be observed. The genericity of our numerical results may ultimately provide a basis for a field theory for non-affine behavior.

### Actin Cytoskeletal Mechanics

Our results not only contain important mathematical implications for rigidity percolation, but important biological implications for the actin cytoskeleton. Actin cytoskeletal networks are typically compositely crosslinked (with presumably even more than two types of crosslinkers). To be concrete, we propose that alpha-actinin as a candidate for a freely-rotating crosslinker, particularly since this property has been observed in optical trapping experiments of actin filaments crosslinked with alpha-actinin [5]. As for an angle-constraining crosslinker such that there exists an energy cost for rotating two crosslinked actin filaments with respect to each other, we conjecture that filaminA (FLNa) is a possible candidate at small strains. This claim is supported by a recent model for FLNa where the natural resting angle between filaments is approximately 90 degrees [9], [10]. In addition, experiments in the linear strain regime for networks crosslinked with both alpha-actinin and FLNa find that the modulus is much higher at the same total crosslinker concentration than for purely alpha-actinin crosslinked networks [39]. This result not only demonstrates the cooperativity of 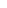-actinin and FLNa working to enhance the mechanical stiffness of actin networks, but also suggests that the two crosslinkers affect the mechanics differently, corroborating our model and findings. Moreover, our conjecture is in good agreement with the experimental observation that FLNa creates an F-actin network at filament concentrations lower than any other known crosslinker [9], [10] since the addition of angle-constraining crosslinkers (with any resting angle) lowers the onset of rigidity in filamentous networks.

**Figure 6:**
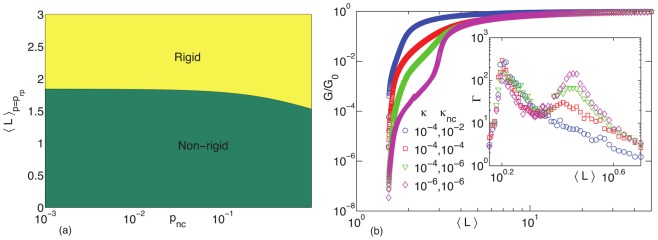
Phase boundary and shear modulus as a function of average filament length. Left: The phase boundary between non-rigid and rigid, as defined by the minimum average filament length for which the shear modulus is nonzero, as a function of the probability of angle-constraining crosslinkers being present (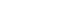 and 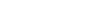). Right: The shear modulus (normalized by the shear modulus for the undiluted lattice, 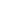) as a function of the average filament length, 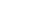 for the same values of 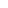 and 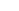 in Fig. 2(c). The inset plots the non-affinity parameter 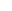 as a function of 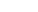 for, again, for the same values of 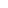 and 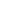 in Fig. 2(c).

Let us delve into the important biological implication of this last result. By tuning the concentration of FLNa, for example, the cell can modulate the minimum concentration of actin filaments necessary to attain mechanical rigidity. Figure 6(a) shows the phase boundary between rigid and not-rigid phases as a function of the angle-constraining crosslinker concentration which, in turn, lowers the average filament length required for rigidity. Most importantly, the threshold average filament length can be essentially as low as the average filament length required to form a geometrically percolating structure, which is the lower bound. When the onset of mechanical rigidity is very close to the geometric percolation threshold, the system is optimizing for rigidity with the least amount of material. Such an optimization principle is very reasonable given the finite amount of scaffolding material in the cell. We have now mathematically justified it with an effective medium theory calculation and numerical simulations for the first time. Note that this result is independent of the energy cost for rotating to crosslinked actin filaments as well as size of the crosslinker.

Is there a way to estimate the energy cost for rotations between crosslinked filaments? For generic actin networks, the ratio of bending rigidity to extensional modulus of an individual actin filament is 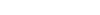 [27], [28]. In Figure 6(b) we plot the shear modulus as function of average filament length for different ratios of 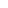 to 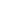. We observe a vanishing of the bend-stretched coupled regime as 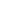 is increased beyond 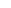 Since the bend-stretch coupled regime has not been observed in prior experiments on *in-vitro* actin networks crosslinked with FLNa, we conjecture that the energy cost of deformation of angles between filaments crosslinked with FLNa is larger than the bending energy of filaments, though we should also note that such experiments were performed in the nonlinear regime. In addition, a composite network can suppress the non-affine fluctuations near the isostatic point by increasing the shear modulus of the network and giving rise to a more affine mechanical response while keeping the filament concentration and the total crosslinker concentration fixed. We see this effect in the inset of Figure 6(b) where the non-affinity decreases with increasing 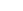 A non-affine response by the network may not always be favorable depending on the perturbation involved.

In addition to the cooperative interplay between alpha-actinin and FLNa allowing the system to easily modulate its mechanical result even at a fixed filament concentration, we observe redundancy in the different types of crosslinked networks, the most important being that the various regimes of elasticity–the bending-dominated regime at small average filament lengths, followed by a bend-stretch dominated regime (for 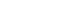 and 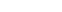) as the average filament length is increased, followed by a stretch-dominated regime. This trend is typically in all three of the systems studied–freely-rotating crosslinked networks, and in both compositely crosslinked flexible and semiflexible filamentous networks, thereby suggesting a built-in redundancy. This result is an indication of the robustness of these networks and should not be considered as a weakness. Whether or not this robustness extends to systems experiencing higher strains such that nonlinearities emerge is not yet known.

### Lamellipodia Mechanics

The interplay between cooperative and redundant mechanical properties may be particularly important for the mechanics of branched F-actin networks in lamellipodia, which are a specialized form of actin cytoskeletal networks. Within lamellipodia, there exist some filament branches occurring at an angle of around 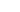 with respect to the plus end of the mother filament (referred to as 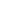 junctions). These branches are due to the ABP Arp2/3 [15] and are presumed to be the dominant channel for filament nucleation. The mechanics of Arp2/3 can be modeled as an angular spring between the mother and daughter filament with an angular spring constant of approximately 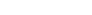 [15]. In other words, Arp2/3 is an angle-constraining crosslinker for 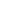
junctions (as opposed to 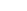
junctions), and thereby plays an important role in lamellipodia mechanics as demonstrated in this work. The mechanical role of Arp2/3 in lamellipodia has not been investigated previously and may help to discriminate between the dendritic nucleation model [13], [14] and a new model for lamellipodia formation [47] by predicting the force transmitted in lamellipodia as a function of the Arp2/3 concentration.

In addition to Arp2/3, FLNa localizes at 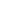
junctions in the lamellipodia and is thought to stabilize the dendritic network [48]. Both angle-constraining crosslinkers lower the filament concentration threshold required for mechanical rigidity in the system. Depending on the energy scale of FLNa as compared to the energy scale of Arp2/3, addition of the FLNa may or may not modulate, for example, the bend-stretch coupling regime at intermediate filament concentrations. Again, at times mechanical redundancy is needed and at times not. With three crosslinkers, the system can maximize the redundancy and the cooperativity. Of course, lamellipodia are dynamic in nature and are anisotropic since the Arp2/3 is activated from the leading edge of a cell. Both attributes will modulate the mechanical response.

### Tissue Engineering

While our focus has been mostly on cytoskeletal mechanics, our results are also relevant for collagen and fibrin networks. Both networks can be used as potential scaffolds for tissue engineering [18], [19]. The somewhat regular branching angle between filaments in these networks suggests that there is an energy cost of deforming the angle between filament branches such that our model applies. The simultaneous presence of angle-constraining and freely-rotating crosslinks enables the network to attain rigidity and transmit forces just above the geometric percolation threshold allowing the scaffold to be maximally porous. Upon further addition of crosslinkers, the strength of the scaffold can be increased by several orders of magnitude, presumably enough to support growing cells. Indeed, the study of angle-constraining crosslinks in filamentous networks may aid in designing very porous, yet very strong biological scaffolds needed for tissue engineering.

In closing, we have demonstrated both cooperativity and redundancy in the mechanics of compositely crosslinked filamentous networks. We have done so while maintaining the structure of an isotropic, unbundled filament network. Of course, crosslinkers can also alter the morphology of the network via bundling, for example. This change in microstructure will presumably affect the mechanics such that the cooperative and redundant interactions between multiple types of crosslinkers will differ from the above analysis and should ultimately be investigated theoretically. In this study, however, we find both cooperativity and redundancy in the network mechanics even in the absence of such structural changes [49], which, is arguably less intuitive and, therefore, more remarkable. Since the cytoskeleton consists of a finite amount of material, the ability to alter mechanics without introducing major morphological changes or motifs may play important role in processes such as cell motility and shape change.

## Supporting Information

File S1 This file contains more calculational details for the effective medium theory.
(PDF)
