# Supplementary material for: Redundancy and Cooperativity in the Mechanics of Compositely Crosslinked Filamentous Networks
Source: PLoS One. 2012 May 9;7(5):e35939. doi: 10.1371/journal.pone.0035939 (PMC3348909; doi:10.1371/journal.pone.0035939)
Supplement: File S1 — This file contains more calculational details for the effective medium theory. (PDF) [file pone.0035939.s001.pdf]

I. SUPPORTING INFORMATION

A. Effective medium theory: Derivation of the effective medium elastic moduli

Here we describe the basic principles in the construction of our effective medium framework and the derivation of the effective medium elastic constants for filament stretching, filament bending, and stiffness of angular crosslinks of the disordered networks under consideration. The central assumption in our effective medium theory is that strain fluctuations produced in the original, ordered network by randomly cutting filaments and removing angular springs vanish when averaged over the entire network.

Let us consider an ordered network with each bond having a spring constant μ_m, a filament bending constant for adjacent collinear bond pairs κ_m, and an angular bending constant $\kappa_{nc,m}$ between bonds making 60° angles. Under small applied strain, the filament stretching and filament bending modes are orthogonal, with stretching forces contributing only to deformations along filaments ($\boldsymbol{u}_{\|}$) and bending forces contributing only to deformations perpendicular to filaments ($\boldsymbol{u}_{\perp}$), and hence we can treat them separately. The angular forces due to the angular (non-collinear) springs, when present, contribute to stretching of filaments as discussed in the Section on Models and Methods in the manuscript, where we only consider three body interactions. For these springs to contribute to bending one needs to consider four-body interactions which is outside the scope of this paper and will be addressed in future work.

We start with the deformed network and replace a pair of adjacent collinear bonds with bending rigidity κ_m by one with a rigidity κ, and a bond spring with extensional elastic constant μ_m by a spring with an elastic constant μ and the facing 60° angular spring by κ_{nc}. This will lead to additional deformation of the above filament segments and the angle which we calculate as follows. The virtual force that needs to be applied to restore the nodes to their original positions before the replacement of the bonds will have a stretching, a bending and an angular contribution: F_s, F_b, and F_θ. The virtual stretching force is given by $F_s = (\mu_m - \alpha - 3\kappa_{nc}/2)u_{\|,m}$, the virtual filament bending force is $F_b = (\kappa_m - \kappa)u_{\perp,m}$, while the virtual force to restore the angle is $F_\theta = (\kappa_{nc,m} - \kappa_{nc})\theta_m$, where $u_{\|,m}$, $u_{\perp,m}$ and θ_m are the corresponding deformations in the ordered network under the applied deformation field. By the superposition principle, the strain fluctuations introduced by replacing the above bending hinges and bonds in the strained network are the same as the extra deformations that result when we apply the above virtual forces on respective hinges and segments in the unstrained network. The components of this "fluctuation" are, therefore, given by:

$$\begin{aligned} d\ell_{\|} &= \frac{F_s}{\mu_m/a^* - \mu_m + \alpha + (3/2)\kappa_{nc}} \\ d\ell_{\perp} &= \frac{F_b}{\kappa_m/b^* - \kappa_m + \kappa} \\ d\theta &= \frac{F_\theta}{\kappa_{nc,m}/c^* - \kappa_{nc,m} + \kappa_{nc}} \end{aligned} \tag{1}$$

The effective medium spring and bending constants, μ_m, κ_m and $\kappa_{nc,m}$, respectively, can be calculated by demanding that the disordered-averaged deformations $\langle d\ell_{\|}\rangle$, $\langle d\ell_{\|}\rangle$, and $\langle d\theta\rangle$ vanish, i.e. $\left\langle \frac{\mu_m - \alpha - 3\kappa_{nc}/2}{\mu_m/a^* - \mu_m + \alpha + 3\kappa_{nc}/2} \right\rangle = 0$, $\left\langle \frac{\kappa_m - \kappa}{\kappa_m/b^* - \kappa_m + \kappa} \right\rangle = 0$, and $\left\langle \frac{\kappa_{nc,m} - \kappa_{nc}}{\kappa_{nc,m}/c^* - \kappa_{nc,m} + \kappa_{nc}} \right\rangle = 0$. To perform the disorder averaging, since the stretching of filaments is defined in terms of spring elasticity of single bonds α, the disorder in filament stretching is given by $P(\alpha') = p\delta(\alpha' - \alpha) + (1-p)\delta(\alpha')$. Filament bending, however, is defined on pairs of adjacent collinear bonds with the normalized probability distribution $P(\kappa') = p^2\delta(\kappa' - \kappa) + (1-p^2)\delta(\kappa')$. Similarly, for the angular springs, the normalized probability distribution is given by $P(\kappa'_{nc}) = p_{nc}p^2\delta(\kappa'_{nc} - \kappa_{nc}) + (1 - p_{nc}p^2)\delta(\kappa'_{nc}))$. This disorder averaging gives the effective medium elastic constants as a function of p and p_{nc} as

$$\begin{aligned} & p^3 p_{nc}\left(\frac{\mu_m - \alpha - 3\kappa_{nc}/2}{\mu_m/a^* - \mu_m + \alpha + 3\kappa_{nc}/2}\right) + (1-p)p^2 p_{nc}\left(\frac{\mu_m - 3\kappa_{nc}/2}{\mu_m/a^* - \mu_m + 3\kappa_{nc}/2}\right) \\ + \;& p(1 - p^2 p_{nc})\left(\frac{\mu_m - \alpha}{\mu_m/a^* - \mu_m + \alpha}\right) + (1-p)(1-p^2 p_{nc})\left(\frac{\mu_m}{\mu_m/a^* - \mu_m}\right) = 0 \\ & \frac{\kappa_m}{\kappa} = \frac{p^2 - b^*}{1 - b^*}, \text{ and } \frac{\kappa_{m,nc}}{\kappa_{nc}} = \frac{p_{nc}\; p^2 - c^*}{1 - c^*}. \end{aligned} \tag{2}$$

The constants a^*, b^* and c^* for the network contribution to the effective spring constant μ_m/a^* of bonds, to the filament bending rigidity κ_m/b^*, and the bending rigidity κ_{nc}/c^* of angular springs making 60° angles respectively, are

given by $a^*, b^*, c^* = \frac{2}{Nz}\sum_q Tr\left[\boldsymbol{D}_{s,b,nc}(q)\boldsymbol{D}^{-1}(q)\right]$. The sum is over the first Brillouin zone and z is the coordination number. The stretching, filament bending and non-collinear bending contributions, $\boldsymbol{D}_{s,b,nc}(q)$ respectively, to the full dynamical matrix $\boldsymbol{D}(q) = \boldsymbol{D}_s(q) + \boldsymbol{D}_b(q) + \boldsymbol{D}_{nc}(q)$, are given by:

$$\begin{aligned}
\boldsymbol{D}_s(q) &= \mu_m \sum_{\langle ij\rangle}\left[1 - e^{-i\boldsymbol{q}.\boldsymbol{r}_{ij}}\right]\boldsymbol{r}_{ij}\boldsymbol{r}_{ij} \\
\boldsymbol{D}_b(q) &= \kappa_m \sum_{\langle ij\rangle}\left[4(1-\cos(\boldsymbol{q}.\boldsymbol{r}_{ij}))\right. \\
&\quad \left. -(1-\cos(2\boldsymbol{q}.\boldsymbol{r}_{ij}))\right](\boldsymbol{I} - \boldsymbol{r}_{ij}\boldsymbol{r}_{ij}) \\
\boldsymbol{D}_{nc}(q) &= \frac{3}{2}\kappa_{nc,m}\sum\left[2(1-\cos(\boldsymbol{q}.\boldsymbol{r}_{ij})) + 2(1-\cos(\boldsymbol{q}.\boldsymbol{r}_{ik}))\right. \\
&\quad \left. -2(1-\cos(\boldsymbol{q}.\boldsymbol{r}_{jk}))\right]\boldsymbol{r}_{ij}\boldsymbol{r}_{ik}
\end{aligned} \tag{3}$$

with $\boldsymbol{I}$ the unit tensor and the sums are over nearest neighbors. Note that for small q, $\boldsymbol{D}_b \sim q^4$ and $\boldsymbol{D}_s \sim q^2$ have the expected wavenumber dependencies for bending and stretching.

Finally, in constructing the effective medium theory we have assumed that any bending hinge, collinear or non-collinear, can be replaced independently of its neighboring hinges. While this represents an uncontrolled approximation since on removing a bond, both bond pairs containing that bond would be affected, the disorder distribution for the bending rigidity defined on a given bond pair is rigorously correct. For a given bond pair ijk the bending rigidity is zero when (i) only one of the two bonds ij and jk is absent, with a probability $p(1-p)$ for either, (ii) both are absent, with a probability $(1-p)^2$. This gives a total probability $(1-p)^2 + 2p \times (1-p) = 1 - p^2$ for the above bond pair to have a zero bending rigidity. On the other hand, it has a non-zero bending rigidity κ when both bonds are present, with a probability p^2. Since bending is defined on pairs of adjacent bonds and not single bonds in the deformation energy, we speculate that effective medium theories that do not take into account such a "double-bond" distribution for the disorder in bending may not be able to accurately capture the rigidity percolation thresholds for filament bending or bond bending networks. While the "double-bond" assumption leads to an overestimation of the contribution of collinear and non-collinear bending elasticity to the elastic moduli for sparse networks that are bending dominated, it seems to essential for incorporating the correct disorder distribution for bending within a simple effective medium framework. Furthermore, since for small deformations, bending and stretching of filaments are decoupled, the disorder averaging for bond stretching is not affected by this assumption. Finally, as can be seen in our Results, the rigidity percolation thresholds and the different mechanical regimes we obtain are in excellent agreement with numerical simulations in our manuscript and prior numerical simulations.
